# Complete Genome Sequence of the Pathogenic *Vibrio vulnificus* Type Strain ATCC 27562

**DOI:** 10.1128/genomeA.00907-17

**Published:** 2017-08-31

**Authors:** Douglas B. Rusch, Dean A. Rowe-Magnus

**Affiliations:** a Center for Genomics and Bioinformatics, Indiana University Bloomington, Bloomington, Indiana, USA; b Department of Biology, Indiana University Bloomington, Bloomington, Indiana, USA; c Molecular and Cellular Biochemistry, Indiana University Bloomington, Bloomington, Indiana, USA

## Abstract

*Vibrio vulnificus* has the highest death rate and economic burden per case of any foodborne pathogen in the United States. A complete genome sequence of the type strain promotes comparative analyses with other clinical and environmental isolates, improving our understanding of this important human pathogen and successful environmental organism.

## GENOME ANNOUNCEMENT

*Vibrio* species cause 80,000 cases of vibriosis and 100 deaths in the United States each year (see www.cdc.gov/vibrio/index.html). *Vibrio vulnificus* causes the majority of vibriosis-related deaths. Infection can occur via the exposure of open wounds to contaminated water or through the ingestion of contaminated seafood. *V. vulnificus*, unlike other vibrios, causes septicemia (1), and infection outcomes can be grim (disfigurement, amputation, or death) and costly. It has the highest hospitalization (>90%) and death (>50% for septicemic patients) rates of any foodborne disease agent, and the economic burden per case ($3.3 million) is highest among foodborne pathogens (2). A complete genomic picture is needed to understand the mechanisms governing *V. vulnificus* pathogenesis and evolution and to pursue strategies to decrease *Vibrio* loads in shellfish and reduce the incidence of invasive disease.

The previous draft genome sequence of 194 contigs lacked key regulatory (*smcR*, *polB*, and *rho*), DNA repair (*mutL*, *recBCD*, and *uvrC*), or amino acid and vitamin biosynthesis genes (histidine, tryptophan, thiamine, biotin, and cellobiose operons). Moreover, important repeat-containing regions, such as the notoriously difficult-to-assemble chromosomal superintegron (SI) (3, 4), were fragmented. We present here the complete genome sequence of the type strain. Single-end Illumina NextSeq 150-bp reads (14 M) were quality and adapter trimmed (Trimmomatic-0.30 [5], with 97.5% of the reads surviving the trimming process). Additionally, 37,373 PacBio reads were corrected using PacBioToCA (6) (total length of 127 Mbp, ~25-fold coverage of the genome). The genome was assembled from a combination of PacBio and Illumina reads with the Celera assembler (version 8.1) (7, 8). This yielded two large contigs corresponding to the two anticipated chromosomes, which showed strong synteny to previously sequenced complete genomes (9, 10). The chromosome ends were manually curated for circularization using PacBio reads.

The large chromosome (chromosome 1 [Chr I], 46.5% G+C content) is 3,266,104 bp long and harbors 2,874 predicted coding sequences, 111 tRNAs, and 10 16S-23S-5S rRNA operons. The small chromosome (chromosome 2 [Chr II], 47.1% G+C content) is 1,741,042 bp long and contains 1,428 predicted coding sequences, 13 tRNAs, and one 16S-23S-5S rRNA operon. The type strain has the largest SI identified to date, at 158,319 bp long and 230 gene cassettes. The overall G+C content of the SI was 5.2% lower than the average for the chromosome on which it resides (Chr I), suggesting that most of the gene cassettes are of foreign origin. The majority of open reading frames (ORFs) within the SI had no database homologs, and <40% of the gene cassettes shared similarity (maximum E value, 1e^-3^) with the 554 collective gene cassettes from the SIs of strains MO6-24/O, YJ016, and CMCP6, supporting the important role of this recombination system in bacterial genome evolution (4, 11, 12).

### Accession number(s).

The sequence has been deposited in GenBank under accession numbers CP012881 and CP012882.
